# Metformin Affects Heme Function as a Possible Mechanism of Action

**DOI:** 10.1534/g3.118.200803

**Published:** 2018-12-15

**Authors:** Xiyan Li, Xin Wang, Michael P. Snyder

**Affiliations:** Department of Genetics, Stanford University, Stanford, CA 94305-5120

**Keywords:** metformin, heme, hemoprotein, diabetes, porphyrin, mechanism of action, redox

## Abstract

Metformin elicits pleiotropic effects that are beneficial for treating diabetes, as well as particular cancers and aging. In spite of its importance, a convincing and unifying mechanism to explain how metformin operates is lacking. Here we describe investigations into the mechanism of metformin action through heme and hemoprotein(s). Metformin suppresses heme production by 50% in yeast, and this suppression requires mitochondria function, which is necessary for heme synthesis. At high concentrations comparable to those in the clinic, metformin also suppresses heme production in human erythrocytes, erythropoietic cells and hepatocytes by 30–50%; the heme-targeting drug artemisinin operates at a greater potency. Significantly, metformin prevents oxidation of heme in three protein scaffolds, cytochrome c, myoglobin and hemoglobin, with Kd values < 3 mM suggesting a dual oxidation and reduction role in the regulation of heme redox transition. Since heme- and porphyrin-like groups operate in diverse enzymes that control important metabolic processes, we suggest that metformin acts, at least in part, through stabilizing appropriate redox states in heme and other porphyrin-containing groups to control cellular metabolism.

Metformin is a biguanide drug that is primarily used at high concentrations to treat hyperglycemia and insulin resistance (He and Wondisford 2015; Miranda *et al.* 2014). It is safe for long-term use and even reduces mortality rates in diabetic patients below those of normal (non-diabetic) people (Campbell *et al.* 2017; Diabetes Prevention Program Research Group 2012). Despite its remarkable role in gluconeogenesis inhibition and pleiotropic health benefits beyond diabetes, metformin’s mode of action at the molecular level is not well understood. Previous studies have reported that metformin acts through multiple different protein targets, including the mitochondrial complex I in the electron transport chain (ETC) (Wheaton *et al.* 2014), an intracellular energy starvation responder AMP-activated kinase (*AMPK*) (Zang *et al.* 2004), an organic cation entry gateway OCT1 (Chen *et al.* 2014), a succinate G-protein-coupled receptor (Guo *et al.* 2017a), a mitochondrial glycerophosphate dehydrogenase (Madiraju *et al.* 2014), and glucagon receptor-mediated *PKA* signaling (Miller *et al.* 2013). The functions of many of these targets are connected with mitochondria and even the gut microbiota (Hur and Lee 2015). However, metformin’s action on most of these targets either appears to be mediated through indirect interactions (Miller *et al.* 2013) or may represent a minor route of action (He and Wondisford 2015). Thus it remains unclear whether other mechanisms exist and/or whether a unifying or dominating mechanism can explain metformin action.

From a biochemical perspective, several lines of evidence point to a possible mode of intracellular action of metformin. First, metformin is not metabolized in humans and is excreted rapidly from the plasma without modification (Robert *et al.* 2003), suggesting a direct modulation of molecular function(s). Second, the total daily dosage of metformin in current medical practice ranges from 1000 mg to 2500 mg, and is often taken twice daily resulting in high micromolar to low millimolar concentrations in extracellular body fluids (He and Wondisford 2015). Measured plasma metformin can reach as high as 0.85 mM in some individuals (Lalau *et al.* 2011). This observation suggests that metformin either acts with a weak pharmacokinetic potency, or its target is of high abundance, or both. Third, although reports vary widely and early reports suggested low lntracellular concentrations of metforminat 2 μM (Robert *et al.* 2003), more recent reports of the same group indicate that metformin is retained intracellularly at very high concentrations (>200 μM intracellularly *vs.* 5 μM extracellularly) (Lalau and Lacroix 2003) and can be as high as 470 μM in some individuals (Lalau *et al.* 2011). Furthermore, intracellular metformin has a much longer elimination half-life (23.4 h) than in the extracellular metformin (2.7 h) (Chien *et al.* 2016; He and Wondisford 2015; Robert *et al.* 2003), suggesting that intracellular metformin-binding targets are abundant. Among common body cells (hepatocytes and erythrocytes) that have been examined, the distribution of metformin is 3-24 fold higher in the cells than in the plasma, and the clearance time in erythrocytes can be 8.7-fold longer than for plasma (Robert *et al.* 2003; Wilcock *et al.* 1991). Thus, a high effective concentration is needed for medical efficacy. These findings together appear to support the concept that metformin may act on cellular targets of high abundance and prevalence. Given the ubiquitous and diverse metabolic effects elicited by metformin, which includes lowering blood glucose, impairing tricarboxylic acid cycle (TCA) and suppressing gluconeogenesis, the targets of metformin may bear a common feature among these processes both structurally and functionally.

The enrichment and extended clearance of metformin in erythrocytes are particularly intriguing. Erythrocytes play important functions *en masse* in fueling catabolic metabolism by providing oxygen, which aligns with the functional scope of metformin in modulating aerobic respiration in mitochondria (Janzer *et al.* 2014; Li *et al.* 2015). A major biochemical component for erythrocyte function is hemoglobin and its associated heme groups. Because of its intrinsic chemical function as the oxygen carrier, the ferrous ion in the center of heme is subject to spontaneous oxidation to ferric state, which does not bind oxygen and accounts for 1–2% of total heme even in normal conditions (Lata and Janardhanan 2015). To salvage the ferric heme to ferrous heme, erythrocytes use glucose to regenerate NADPH, which in turn is used to reduce ferric heme (Lata and Janardhanan 2015). These cells lack mitochondria and their energy source is solely dependent upon anaerobic glycolytic breakdown of glucose, with the production of lactate, whose accumulation (lactic acidosis) is a well-known side effect for metformin in patients with defective clearance of metabolic waste (Lalau 2010). Erythrocytes consume 10% of the total blood glucose, which can rise to 1.5 fold when they are stressed by mere mechanic stirring (Kodícek 1986). Energy expenditure at the stimulated level would place erythrocytes as the fourth biggest energy consumer of the body, after liver (27%), brain (19%), and skeletal muscles (18%) (Durnin 1981). Thus, erythrocytes may be a major glucose modulator and a target for metformin through their glucose consumption.

In the present study, we examined potential functional connections between metformin and heme in both yeast and human cells. We found that metformin, at millimolar concentrations, suppressed heme production by over 50% in yeast, which can be abrogated by blocking mitochondrial function. Metformin also reduced hemoglobin oxidation by 50% in human erythrocytes and erythropoietic cells but did not affect cell viability. In human liver cells, metformin suppressed heme production to a similar extent, and another heme-targeting drug artemisinin was even more potent. In a simple cell-free chemistry system, metformin suppressed the redox transition of heme groups in three different proteins: cytochrome c, hemoglobin, and myoglobin, supporting an interaction between metformin and heme containing proteins *in vitro*. Based on these observations we propose a unifying mechanism for metformin action.

## Materials and Methods

### Yeast strains and growth

Several experimental Saccharomyces cerevisiae strains, including a haploid strain *BY4741* (MATa his3Δ1 leu2 Δ0 met15 Δ0 ura3 Δ0), a diploid strain *BY4743* (MATa/α his3 Δ1/his3 Δ1 leu2 Δ0/leu2 Δ0 lys2 Δ/LYS2 MET15/met15 Δ0 ura3 Δ0/ura3 Δ0), and a deletion strain mip1Δ (in the BY4741 background), were used in this study.

The YPD media contained yeast extract (1% w/v), peptone (2%, w/v), dextrose (2% w/v). For the growth assay, an overnight seed culture in YPD media was diluted 100-fold in fresh YPD media, and then mixed with a fixed volume of solution containing different dose of metformin. Yeast cultures were aliquoted and incubated in a Tecan plate reader at 30°C with agitation. Optical density at 600 nm was determined every 15 min during a period of 2 to 3 days. The optical density of end/stationary phase cultures was also determined manually on a UV/visual wavelength spectrometer.

### Heme content measurement

The total heme content in yeast cells was measured by a protocol as described previously (Sinclair *et al.* 2001). Briefly, yeast cells grown in liquid media were collected by centrifugation at 6,000 × g for 5 min, and washed in water once. The cell pellet was weighed and re-suspended and split into two aliquots in microcentrifugation tubes, and pelleted again by centrifugation at 6,000 × g for 5 min. After vortex mixing in 2M oxalic acid, one sample was heated at 95° for 30 min, and the other was placed at room temperature as the “blank”. At the end, both sample were centrifuged again, and the supernatant were measured by fluorescence on a plate reader with the following settings: excitation at 400 nm, emission at 608 nm and 662 nm, dwelling time at 100 ms. The difference between the readings of the “blank” and the heat-treated sample at either wavelength was used as the arbitrary unit for total heme content. The final reported heme content was normalized to the weight of biomass.

A Quantichrom Heme Assay kit from BioAssay Systems (Fisher Scientific 50489220) was used to measure heme content in the human blood or plasma according to the manufacturer’s instructions. Briefly, blood or plasma was diluted 1:10 in the Reagent (no cells in the plasma). After brief mixing, absorption at 400 nm was determined on a spectrometer. Heme content was calculated by comparison to a standard curve.

### Human cell culture and blood

Human myeloid precursor cell line K562 and hepatocyte cell line HepG2 were bought from the American Type Culture Collection (ATCC) and cultured in RPMI 1640 media (Life Technology 31800022) supplemented with 10% fetal bovine serum (Life Technology 10082139) in an incubator set at 37°C and 5% CO_2_. The cells were maintained and passaged in fresh media every 2-3 days until use.

De-identified human blood samples were acquired from the Stanford Blood Center. The erythrocytes were maintained in RPMI 1640 media with 10% fetal bovine serum in an incubator set at 37°C and 5% CO_2_.

### Cell viability assay

Viability assay of cultured human cells was performed in 96-well plates using the CellTiter-Glo luminescence assay (Promega PRG7572), following the manufacturer’s instructions. Briefly, the CellTiter-Glo reagent was added as 1/5 equivalent volume into the culture media and mixed briefly to lyse the cells. Luminescence was measured on a Tecan plate reader using an integration time of 0.25-1s.

### Triglyceride measurement

Triglyceride levels were measured using an Infinity Triglycerides kit (Thermo Scientific TR22421) according to the manufacturer’s instructions. Briefly, cells were gently pelleted and washed in PBS buffer, then lysed in the lysis buffer (20 mM Tris-HCl, pH 8.0, 1% Nonidet P-40, 1 mM EDTA, 1 mM EGTA, supplemented with 1x Halt Protease & Phosphatase Inhibitor Cocktail (Thermo 78446)), and then mixed with the measurement Reagent at a ratio of 1:100. Absorption at 500 nm was determined and the total amount of triglycerides was calculated after normalization to a blank control lacking triglycerides and a calibrator.

### Heme and cytochrome c oxidation measurement

Lyophilized human hemoglobin (MP Biomedicals 0855914), reduced cytochrome c (Abcam ab140219) or human myoglobin (Alfa Aesar AAJ67726MF) was incubated individually at 50 mM with varying amount of metformin in PBS solution with 2 mM NH_4_OAc. For each target, absorption at a specific wavelength after background subtraction was used to measure the level of the reduced form of this protein, upon incubation with metformin at ambient condition for the duration times specified in the text. The specific wavelength for each target molecule was empirically determined by an absorption-wavelength scan from 400 nm to 600 nm.

### NADPH-cytochrome c reductase activity

NADPH-cytochrome c reductase activity in the equine heart was measured, according to the manufacturer’s instructions (Sigma-Aldrich: CY0100-1KT). Briefly, 5 ml of reconstituted NADPH-cytochrome c reductase enzyme solution was mixed with 5 ml of ligand solution that contains varying amount of metformin and 190 ml of working solution. To initiate the reaction, 50 ml NADPH solution at 0.85 mg/ml was added and mixed, and optical absorption at 550 nm was continuously monitored for 2 min at an interval of 10 s. The slope of the absorption curve *vs.* time was used to calculate the enzyme activity by the formula: Units/ml = DA550/min x dilution × 1.1 /21.1 x Enzyme volume.

Strains are available upon request. The authors affirm that all data necessary for confirming the conclusions of the article are present within the article, figures, and tables.

### Data availability

The authors state that all data necessary for confirming the conclusions presented in the article are represented fully within the article.

## Results

### Metformin suppresses yeast growth and heme content at high dosage

We used a diverse set of experiments to investigate the mechanism of action of metformin. We first used yeast, given its simplicity of growth and large bank of genetic mutants, to suggest possible mode(s) of action. We then tested the effects of metformin on human cells for its relevance to humans. Finally, *in vitro* experiments were performed to directly investigate the biochemical effects of metformin action.

**Figure 1:**
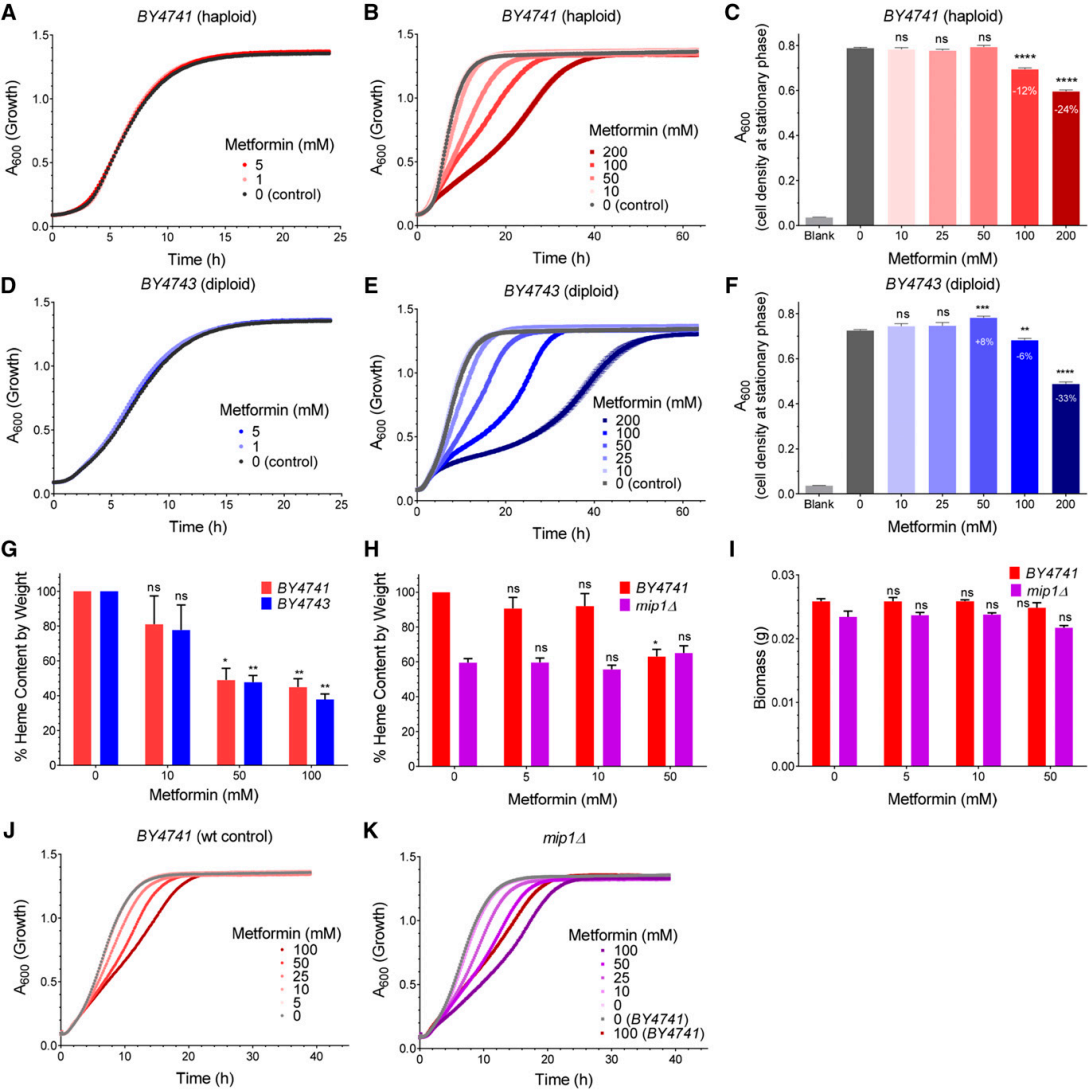
Metformin modulates growth and heme production in yeast. Cell growth was continuously monitored by absorption at 600 nm (A_600_) for the haploid strain *BY4741* (A-C, N = 6) and the diploid strain *BY4743* (D-F, N = 6) cells incubated with metformin at different concentrations from 0-200 mM (A, D) and 0-5 mM (B, E). The endpoint A_600_ after 16 h incubation was determined by a light spectrometer (C, F). The relative heme content of yeast cells after metformin treatment was determined by oxalic acid method (G, N = 3, also see Materials and Methods). The growth of wild-type control (*BY4741*, H) and a mitochondria-deficient mutant strain *mip1Δ* (I) was also affected by metformin (N = 3), and the corresponding relative heme content (J) and biomass (K) were also determined. P values were given for unpaired Welch’s *t*-test. ns = not significant; *, *P* < 0.05; **, *P* < 0.01; ***, *P* < 0.001; ****, *P* < 0.0001. Error bars = SEM.

**Figure 2:**
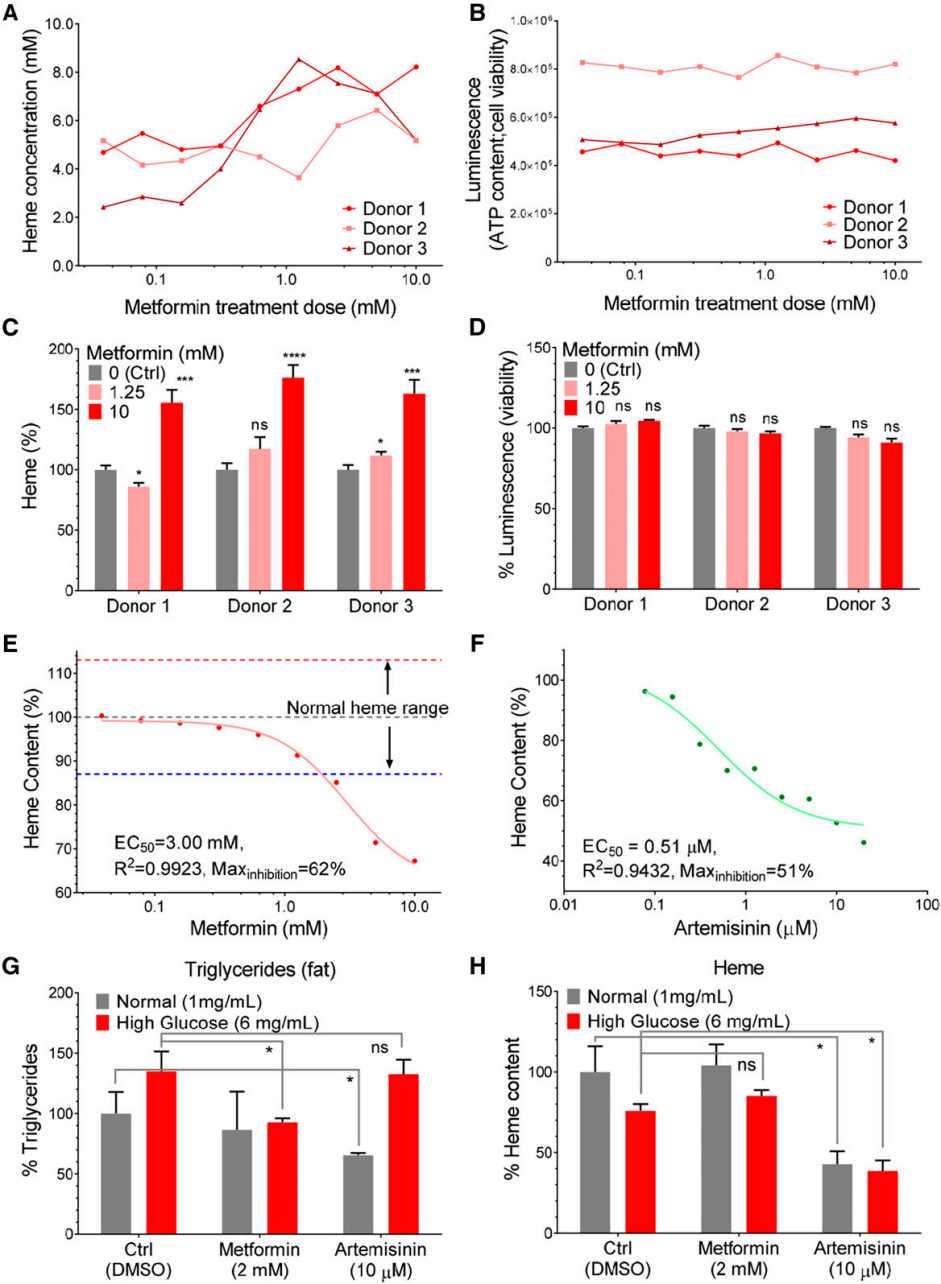
Metformin modulates heme in human cells. The heme content (A) and cell vitality (CellTiter-Glo luminescence) (B) of human erythrocytes were determined after incubation with metformin at varying levels for 24 hr. The relative heme levels (C, technical N = 8) and cell vitality (D, technical N = 4) in human blood cells were compared with unpaired Welch’s *t*-test and the significance was indicated as: ns, not significant; *, *P* < 0.05; **, *P* < 0.01; ***, *P* < 0.001; ****, *P* < 0.0001. The relative heme content in cultured K562 cells, a line of hematopoietic lineage, was determined after treatment with metformin (E) or anti-malaria drug artemisinin (F) for 16 hr, one representative curve from two experiments is shown for each drug. Effective concentration at 50% effect (EC50), correlation coefficient (R^2^) and max inhibition were calculated after curve-fitting to single inhibitor model. The triglyceride (G) and heme levels (H) in human liver cells (HepG2) were determined after metformin and artemisinin treatment under normal and high glucose (6 mg/ml) conditions, P values from unpaired *t*-test were indicated as: ns, not significant; *, *P* < 0.05; for duplicate results (N = 2).

We first determined the concentration of metformin that elicits an effect on yeast growth in rich Yeast extract-Peptone-Dextrose media (YPD). As shown in Figure 1, at 5 mM or below, metformin had no influence on yeast growth when compared with untreated controls in both haploid (*BY4741*) and diploid (*BY4743*) strains (Figure 1A and D). However, in the range of 10 to 200 mM, metformin inhibited growth as evidenced by: 1) the doubling rate of the exponential growth phase was progressively reduced by increasing doses of metformin, resulting in a 12-fold slower growth rate at the highest metformin dose (200 mM) (Figure 1B and E); 2) the optical density of the culture upon reaching stationary phase was also reduced by increasing doses of metformin after a 16-hour incubation (Figure 1C and F). The most pronounced growth inhibition was observed for 200 mM metformin (a 25% growth decrease in *BY4741* and 33% in *BY4743*). Although these doses are high, it is not uncommon for yeast to exclude exogenous compounds (Balzi and Goffeau 1995). Based on the observed effects of metformin on yeast growth, we chose two doses (50 mM and 100 mM) in the subsequent analyses.

To test whether metformin may affect heme production in yeast cells, we adopted a sensitive fluorescence-based method to analyze heme (Sinclair *et al.* 2001) ; this was necessary because heme levels in yeast cells are below the detection limit of conventional quantification methods for assaying blood heme. As shown in Figure 1G, when cells were treated with metformin (50 mM or 100 mM), the total heme content from cells at early stationary phase after 16 h culture was reduced by more than 50% in both haploid and diploid yeast. Because the cell density at 50 mM metformin was either not affected or only minimally affected in either strain (Figure 1C and F), the observation of over 50% reduction in heme content with minimal effect on growth is rather surprising. This may be because the dominant mode of metabolism of yeast in exponential phase is non-heme-requiring anaerobic fermentation (Werner-Washburne *et al.* 1993) ; biomasses would be expected to be less affected than endogenous heme production, a process that requires respiratory metabolism.

In yeast and other eukaryotic cells, heme serves as the electron carrier in cytochrome c and is mainly involved in aerobic metabolism which requires mitochondrial function, and also in the metabolism of fatty acids and sterols in the cytosol (Hoch and Ortiz De Montellano 2001; Kim *et al.* 2012; Russell *et al.* 2009). We next tested if the suppression of heme by metformin is affected by the absence of mitochondrial function. Mitochondria are required for aerobic metabolism and are also known to be involved in heme biosynthesis in yeast (Mense and Zhang 2006). The yeast gene deletion strain *mip1Δ* lacks the only mitochondrial DNA polymerase gene and thus can only proliferate by anaerobic metabolism (Li and Snyder 2016). We observed a strong reduction in the total heme content in *mip1Δ* cells, compared with wild type cells (Figure 1H). Unlike wild type cells the heme content was not further reduced by metformin in *mip1Δ* cells (Figure 1H), indicating that mitochondrial deficiency and metformin operate through the same pathway for the reduction of heme. Despite a strong reduction in the heme content, the biomass was not altered by metformin treatment in both strains (Figure 1I), possibly due to the rich nutrients in the YPD media and the anaerobic fermentation mode of metabolism under our experimental conditions. We also observed more pronounced reduction of logarithmic phase growth in *mip1Δ* than in the control strain (*BY4741*) at the same metformin concentrations (Figure 1J and K), suggesting that the lack of mitochondria compromised the ability of yeast cells to tolerate metformin, probably by increasing the burden of metformin on other cytosolic targets, some of which may also contain heme, such as catalases and peroxidases (Kathiresan *et al.* 2014; Kirkman and Gaetani 1984; Verduyn *et al.* 1988). Taken together, these results established a connection between metformin and mitochondrial-dependent heme production in yeast.

### Metformin suppresses heme function in human cells

We next tested if metformin exerts similar effects on heme content in human cells. Human erythrocytes cannot synthesize heme *de novo* and lose functional hemoglobin over time due to spontaneous oxidative conversion of heme from its oxygen-binding ferrous state to the non-oxygen-binding ferric state (Lata and Janardhanan 2015). We made several observations regarding heme levels and the effect of metformin on functional heme. First, consistent with previous reports (Hebbel *et al.* 1988), we observed a reduction in heme levels in erythrocytes from three different donors after 24 hr incubation *in vitro* (data not shown). Second, co-incubation of these erythrocytes with varying concentrations of metformin ranging from 0.025 mM to 10 mM, reduced the spontaneous loss of ferrous heme in a dose-dependent fashion (Figure 2A). In these experiments the overall cell viability remained largely unaffected by metformin, as assessed by total cellular ATP content measured with the CellTiter-Glo method (Figure 2B). At 10 mM, metformin helped erythrocytes from the different donors to retain more than 50% ferrous heme from loss due to spontaneous oxidation (Figure 2C), without significant effects on cell viability (Figure 2D). Thus, heme preservation by metformin was not due to increased cell viability. These results indicate that metformin reduces ferrous heme loss due to spontaneous oxidation in human erythrocytes and establishes a functional connection between metformin and hemoglobin maintenance.

To further determine the pharmacological kinetics of metformin effects on heme content, we subjected K562, a proliferative myelogenous cell line that is capable of *de novo* heme synthesis (Andersson *et al.* 1979), to the same concentration range of metformin. Similar to the observation in yeast cells, total heme content in these cultured human cells was reduced by incubation with increasing concentrations of metformin in a dose dependent fashion (Figure 2E). The effective metformin concentration of half-maximal inhibition (EC_50_) was 3 mM, which is close to the hemoglobin levels in erythrocytes (3.9 mM) and estimated effective body fluid metformin concentration in clinical use (see Introduction). The maximal inhibition of heme content in K562 cells by metformin was 38%. Taken together, these results indicated that metformin affects heme content: it reduces heme level in proliferative cells capable of heme synthesis, while it also preserves heme in cells that lack the ability to produce heme. This paradox is addressed in the discussion.

In light of a recent report of the action of artemisinin, another drug with an elusive but broad target spectrum, on heme synthesis (Wang *et al.* 2015), we tested if we could recapitulate the effects of artemisinin on heme content in K562 cells. In the physiologically effective range from 0.08 μM to 20 μM, artemisinin also progressively suppressed the heme content in these cells (Figure 2F). The EC_50_ was determined to be 0.51 μM, consistent with findings of a previous study showing an effective dose of greater than 0.25 μM was required for artemisinin’s anti-malarial activities in human white blood cells (Stocks *et al.* 2007). The maximal inhibition by artemisinin was by 49%, which is modestly higher than that observed for metformin (Figure 2E). Thus, both metformin and artemsisinin appear to affect heme levels in cells.

It has been shown that metformin suppresses triglyceride accumulation under high glucose concentration in cultured human hepatocytes^6^. We therefore tested if artemisinin could also produce similar effect. Similar to previously reported findings, we found that metformin at 2 mM effectively suppressed the increase of triglyceride under high-glucose condition (6 mg/ml, 33 mM) in the human liver cell line HepG2. However, artemisinin at 10 μM was unable to achieve triglyceride suppression (Figure 2G). Instead, artemisinin suppressed triglyceride production by 35% only under normal glucose conditions (1mg/ml, 5.5 mM), which is consistent with our observation that artemisinin has a stronger potency than metformin in the inhibition of heme content *in vitro*. Interestingly, artemisinin also reduced heme content in the Hep2G cells under normal and high glucose conditions, whereas metformin did not rescue the modest reduction of heme content in either glucose conditions (Figure 2H). These results suggest a difference between the modes of action of metformin and artemisinin in their ability to normalize high glucose-induced triglyceride accumulation in cultured human liver cells. It also raised a concern over the safety profile of artemisinin, since this drug may induce severe intracellular triglyceride deficiency, which is consistent with the known side effects of artemisinin to elevate aspartate aminotransferase, a biomarker for liver health (Ribeiro and Olliaro 1998), and informatics suggestion of positive regulation of triglyceride catabolic process by the action of artemisinin-containing herbal medicine (Zhao *et al.* 2016).

### Metformin directly suppressed oxidation of hemoprotein and reduction of cytochrome c

**Figure 3:**
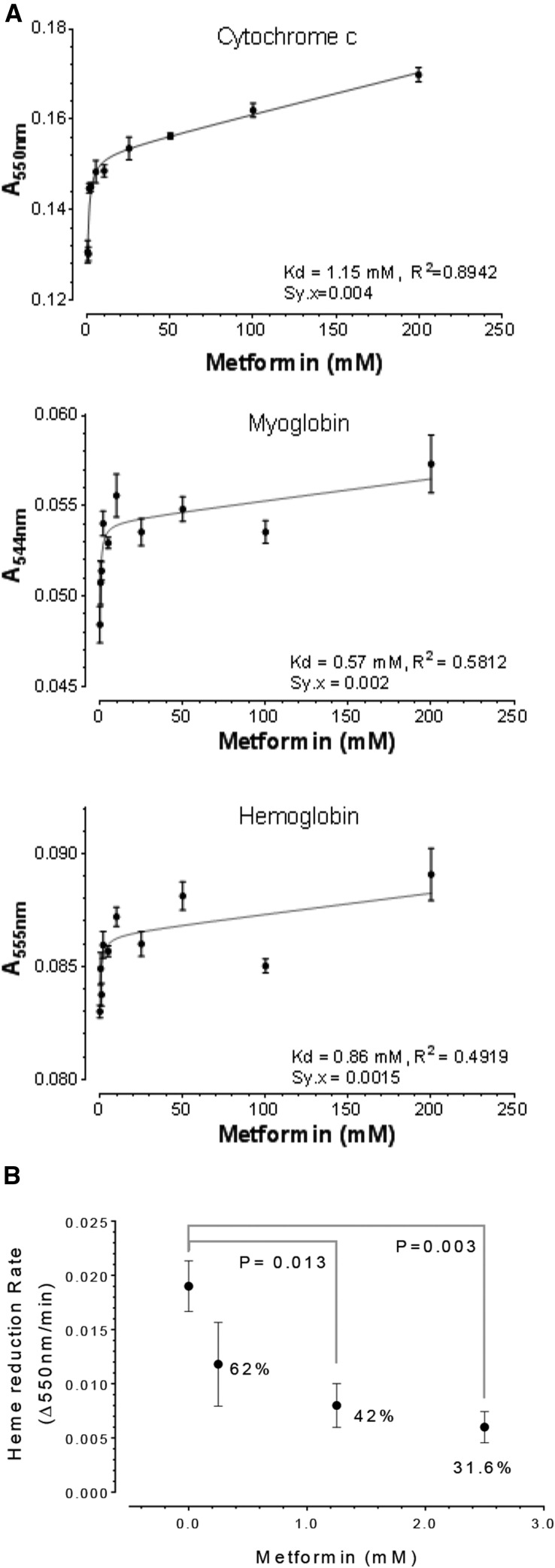
Metformin suppresses heme oxidation and heme-enzyme activity. The absorption at specific wavelength was used to measure the reduced form of cytochrome c, myoglobin, and hemoglobin upon incubation with metformin at ambient conditions for 5, 2, and 1 day(s), respectively (A). Affinity constant (Kd), R^2^, and Sy.x (equivalent to standard error of regression) were based on curve fitting to a one-side binding model, N = 4. The initial enzymatic rate of rabbit liver NADPH-cytochrome c reductase was modulated by metformin (B), P values were from Unpaired Welch’s *t*-test (N = 3-5).

One possible mechanism by which metformin may affect heme content and heme oxidation is by interacting directly with heme-containing proteins; since hemoproteins are in high abundance, this model is consistent with metformin’s high dosage required in clinics (see Introduction). To examine this possibility, we measured the oxidation rate of three hemoproteins, cytochrome c, myoglobin and hemoglobin, in cell-free solutions with varying concentrations of metformin (Figure 3A). We reasoned that, if metformin interacts directly with these hemoproteins, it might protect these proteins from spontaneous oxidation when exposed to air oxygen in a cell-free system, as previously shown^31^. We found that metformin in the range of 0.5 to 200 mM suppressed the oxidation of ferrous heme by measuring ferrous heme-specific absorption (Figure 3A). In particular, for the case of cytochrome c we observed a strong correlative inhibition of oxidation by increasing metformin (R^2^ = 0.8942). A control solution containing only metformin exhibited only background levels of absorption (not shown). The dissociation constant (Kd) for retaining ferrous heme by metformin was 1.15 mM, 0.57 mM and 0.86 mM for cytochrome c, myoglobin and hemoglobin, respectively. Interestingly, these Kd values found in cell-free solutions are close to the effective half-maximal heme inhibition concentration (3 mM) observed for metformin in K562 cells (Figure 2E). Thus from a biochemical perspective, these results demonstrated a direct interaction between metformin and three different hemoproteins with distinctive structures. These proteins’ only common feature is heme, whose oxidation can be attenuated by metformin.

We next reasoned that, should metformin act directly on the heme group, it might affect other biochemical properties of cytochrome c as well. Interestingly, in addition to its ability to prevent cytochrome c oxidation *in vitro*, metformin also inhibited NADPH-cytochrome c reductase and also decreased cytochrome c reduction by 58% at 1.25 mM and by 68% at 2.5 mM (Figure 3B). These observations provide strong support for a direct action of metformin on heme reduction as well. Taken together, our observation indicates that metformin can affect the redox equilibrium state of heme in both oxidation and reduction directions.

## Discussion

Our results suggest that heme and/or similar chemical species is a major molecular target of metformin. Our observation of metformin’s suppression of heme content in cells and its effects on redox transition in both directions (Figures 1-3) is consistent with the reported pharmaceutical profiles of metformin. In all vertebrates, most heme content is found in hemoglobin. Human erythrocytes take up glucose at an initial influx rate of 40 mM/min (Hu *et al.* 2000), which is in turn broken down to re-generate NADPH through the pentose phosphate pathway. The glycolytic product NADPH is mainly used to maintain hemoglobin functions by converting non-oxygen-binding methemoglobin, the ferric heme-carrying form of hemoglobin that is normally produced from spontaneous oxidation of iron, back to ferrous heme-carrying hemoglobin capable of oxygen binding. Even in normal conditions, methemoglobin accounts for 1–2% of total hemoglobin and impairs oxygen delivery by the blood. However, the percentage of methemoglobin can rise to 10–70% in hypoxia conditions and ultimately reach the fatal level at above 70% (Lata and Janardhanan 2015). Given the fact that hemoglobin is the major protein in erythrocytes (250 g/L, or 3.9 mM) and erythrocytes collectively represent a glycolytic biomass 2-3 times the volume present in liver, and that blood comprises the largest internal organ that controls metabolism, the potential of erythrocytes as a major glucose consumer and mobilizer is expected to be significant, yet their influence on blood glucose levels has been largely ignored. As such, the prospect of hemoglobin in the etiology of type 2 diabetes is also intriguing. Considering their large collective mass and overall metabolic demand, erythrocytes may be a substantial glucose consumer when hemoglobin is overwhelmingly challenged by oxidative stress in certain physiological states.

The elevation of blood sugar concentration might be a natural response to maintain erythrocyte function to keep a constant supply of oxygen to other essential organs, especially those with exclusively aerobic metabolism, such as the heart and the brain. Patients with diabetes not only have higher glycosylated hemoglobin HbA1c, they also have elevated reticulocytes (by 47%), the immature form of erythrocytes (Giugliano 1982). This likely reflects a compensatory mechanism that helps to increase glucose consumption in the blood under hyperglycemic conditions. We speculate that this connection is also consistent with the observation that Tibetans show threefold lower prevalence of diabetes than other Chinese ethnic groups (Wang *et al.* 2017), and the Tibetan population carries a high frequency of a hypoxia-inducible factor (*EPAS1*) gene variant that is associated with adaptive lower hemoglobin (Huerta-Sánchez *et al.* 2014; Simonson *et al.* 2010). High altitude-related hypoxia also promotes cardiovascular diseases in pilots with diabetes, who are exposed to occupational risks of hypoxia (Rogers *et al.* 2015). Long-term metformin use is associated with 100% increase in anemia prevalence in diabetic patients (Aroda *et al.* 2016), which might phenocopy Tibetans’ genetic adaptation to high altitude. Protection of heme from oxidative loss may help to maintain normal cellular functions, and particularly, glucose consumption in erythrocytes. These findings and our study together establish a functional connection between metformin, heme and hemaglobin in mammals.

**Figure 4:**
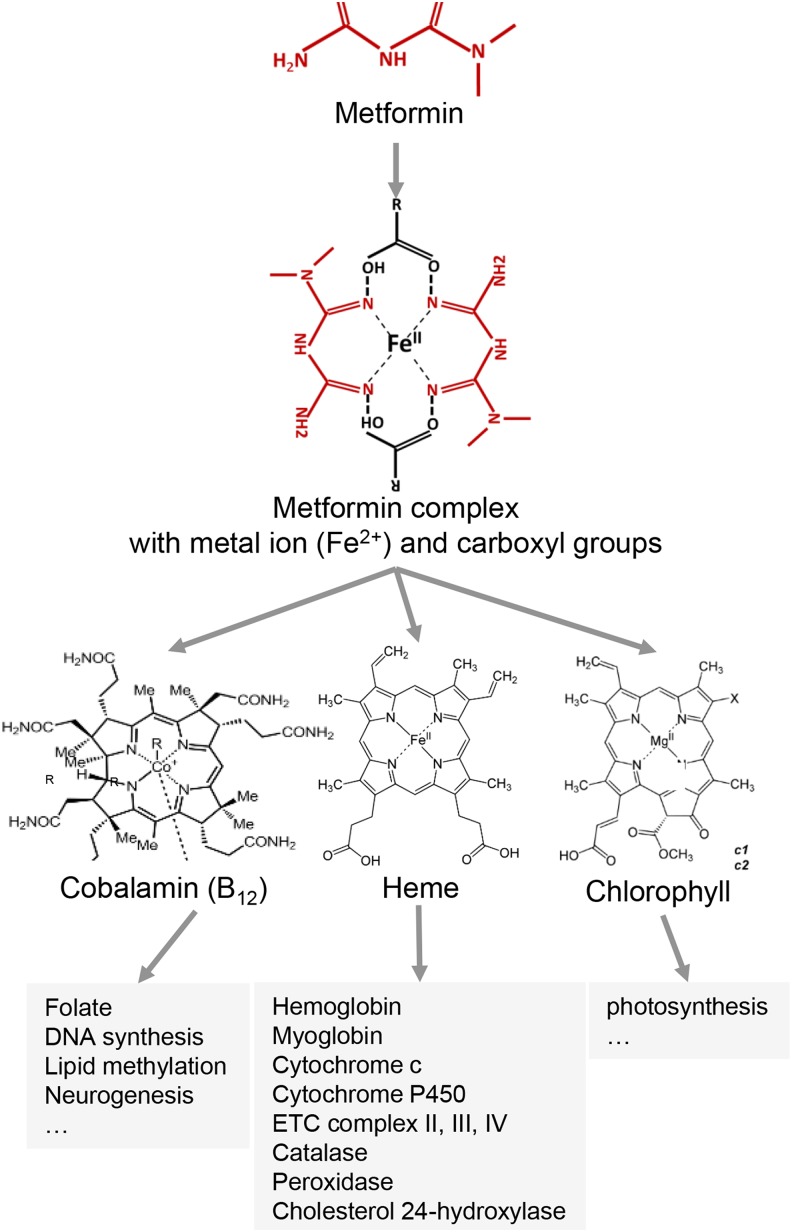
Model for mechanisms of action of metformin. A summary of the mechanism(s) of metformin action is presented.

Overall, direct interaction between metformin and heme-containing groups supports a unifying mechanism that helps to explain the elusive pharmaceutical mode of action of metformin. Metformin itself is capable of sequestering copper in human liver cells and forms a complex with copper *in vitro* (Logie *et al.* 2012; Quan *et al.* 2015). In fact, besides Cu^2+^, metformin also complexes with a broad spectrum of transition-metal ions, such as Co^2+^, Ni^2+^ and Zn^2+^ (and presumably with Fe^2+^ and Mg^2+^ although these were not tested), in the stoichiometry of 1 ion to 2 metformin (Abu-el-wafa *et al.* 1987). These prosthetic ions, when embedded in porphyrin-like structures, mediate reducing/oxidizing biochemical reactions in many important proteins, such as hemoglobin, myoglobin, cytochromes c and P450, catalase, peroxidase, mitochondrial electron transport chain complexes II, III and IV, cobalamin (vitamin B_12_), methane-producing cofactor F430 and chlorophyll-containing cytochrome b6f complex. In all these proteins, the structures of the porphyrin-metal ion complexes completely or partially resemble those of the metformin-metal ion coordinate complexes (Nanubolu *et al.* 2013) (Figure 4); we propose this is the molecular basis for metformin action and its broad-spectrum interventional effects, as demonstrated for heme in human erythrocytes and liver cells, as well as hemoglobin, myoglobin and cytochrome c *in vitro* in this study. Specifically, by forming the coordinate complexes with different free or prosthetic metal ions, metformin may interfere with the activities of proteins containing respective metal coordinate complexes.

The pervasiveness of metformin-targetable functions has likely confounded the elucidation of the mechanism(s) underlying metformin-related effects. For example, although originally derived from plants, metformin suppresses plant growth and development at 6-10 mg/kg soil concentrations (Eggen *et al.* 2011), which, according to our hypothesis, may be explained by its interference with photosynthesis, a chlorophyll-facilitated process. Coincidently, we also found that at high concentrations metformin suppresses heme production in the yeast *Saccharomyces cerevisiae* (Figure 1), an organism previously thought to be insensitive to metformin. We note that yeast does not take up many exogenous drugs, due to its impermeable cell wall and the use of high concentration to explore drug action in yeast is common(Balzi and Goffeau 1995). These observations and our study suggest that a conserved molecular mechanism of metformin action on heme-like groups exists among diverse organisms. The anti-diabetic effects of metformin are believed to be mainly due to inhibition of gluconeogenesis by this biguanide molecule. Earlier studies have suggested that, possibly through its interaction with the mitochondrial complex I (the only complex that does not contain heme), metformin can inhibit mitochondrial activity and increase AMP/ATP ratio, and thus activate *AMPK* to inhibit gluconeogenesis in the liver (Zang *et al.* 2004). Regarding its anti-cancer activity, it has been reported that metformin, by interfering with complex I, inhibits the oxygen consumption rate and growth in human colon cancer cells (Wheaton *et al.* 2014). However, despite this discovery many years ago, direct evidence for metformin-complex I interaction is still lacking, and moreover, it has been shown that *AMPK* is not required for metformin to produce its anti-diabetic effects (Foretz *et al.* 2010; Miller *et al.* 2013). Conversely, the observed effects could result indirectly from metformin suppression of the heme binding protein ferrochelatase, an enzyme that carries out the final step in heme biosynthesis, and is associated only with complex I among all electron transport chain complexes in bovine hearts (Taketani *et al.* 1986). In mammals, a recent study reported that metformin suppresses gluconeogenesis by directly inhibiting the mitochondrial glycerol 3-phosphate dehydrogenase (*mGPD*, aka *GPD2*), an enzyme that converts glycerol into a fuel substrate for gluconeogenesis (Madiraju *et al.* 2014). However, the mGPD enzyme activity assay described in this report is complicated by two factors: 1) the authors used the reduction of cytochrome c as the activity reporter–we showed that as cytochrome-c is a heme-containing protein, the effect is likely directly on cytochrome c rather than *mGPD*; 2) potential contamination of residual mitochondria in *mGPD* isolation through immunoprecipitation in human HEK 293 cells. For instances, *mGPD* is physically located next to all three heme-containing mitochondrial electron transport chain complexes, namely II, III and IV (Mráček *et al.* 2013), therefore cross contamination of these complexes is likely through immunoprecipitation with crude mitochondrial lysate. Finally, we also noted that metformin has been associated with vitamin B_12_ (cobalamin) deficiency in treating type 2 diabetes in placebo-controlled clinical trials and population health survey (de Jager *et al.* 2010; Kibirige and Mwebaze 2013), which could be due to metformin interference with cobalamin, another heme-like porphyrin-containing compound. Interestingly, B_12_ deficiency has been suggested to lead to anemia, a long-term side effect of metformin use in diabetes treatment (Aroda *et al.* 2016; Callaghan *et al.* 1980).

The observation of effective concentrations of metformin in millimolar ranges in this study is consistent with functional studies (Guo *et al.* 2017b). Metformin inhibits breast tumor growth with an IC50 at 5 mM and a cytochrome P450 enzyme activity with Kd50 at 1.5-4.5 mM. Co-crystallization of metformin and the truncated heme-containing enzyme places metformin on the same side of heme with oxygen in hemoglobin (Guo *et al.* 2017b; Terrell *et al.* 2018). These results support a pharmacological model of metformin where the administrated levels (μM) are far below its optimal kinetic levels (mM), which might be consistent with the extraordinary safety profile of metformin for long-term use (Diabetes Prevention Program Research Group 2012).

The metformin suppression on heme-containing proteins may also result indirectly from transcription factor-mediated signaling events through modifying heme biosynthesis. For example, *FOXO3* is a transcription factor required in erythropoiesis to maintain robust control of oxidative stress in mature erythrocytes (Marinkovic *et al.* 2007). Furthermore, metformin relies on *FOXO3* to induce hemoglobin production in primary erythroid cells (Zhang *et al.* 2018). Both studies suggest that metformin may affect heme indirectly through alternative routes.

To conclude, we propose that metformin forms complexes with metal ions that bears structural resemblance to the prosthetic groups in a wide range of important proteins, and thus, interferes with their redox transition and normal functions (Figure 4). Re-examination and expansion of metformin action studies on these proteins may provide new perspective and insights into understanding how metformin functions and guide its clinical applications in treating diabetes and other diseases and even aging (clinical trial NCT02432287) (He and Wondisford 2015; Miranda *et al.* 2014).
